# Acute myocardial infarction and arterial embolism in a patient with newly diagnosed renal mass: management dilemmas! A case report

**DOI:** 10.1186/s12894-021-00870-z

**Published:** 2021-08-18

**Authors:** Benedikt Martin, Andreas Greinacher, Robin Bülow, Fabian Hammer, Andreas Hoene, Martin Burchardt, Uwe Zimmermann

**Affiliations:** 1 Department of Urology, University Medicine Greifswald, Ferdinand-Sauerbruch-Str., 17475 Greifswald, Germany; 2 Department of Transfusion Medicine, University Medicine Greifswald, Ferdinand- Sauerbruch-Str., 17475 Greifswald, Germany; 3 Department of Cardiology, KMG Clinic Guestrow, Friedrich-Trendelenburg-Allee 1, 18273 Guestrow, Germany; 4 Department of Vascular Surgery, University Medicine Greifswald, Ferdinand-Sauerbruch- Str., 17475 Greifswald, Germany; 5 Department of Radiology, University Medicine Greifswald, Ferdinand-Sauerbruch-Str., 17475 Greifswald, Germany

**Keywords:** Acute arterial thrombotic emboli, Paraneoplastic syndrome, Clear cell renal cell carcinoma

## Abstract

**Background:**

Cancer is often associated with a hypercoagulable state and new thrombosis is often the first clinical manifestation of cancer. Surgical treatment of the primary tumor is crucial since it provides the only curative approach in most cases, but management of patients is highly complex, especially in the presence of new antiplatelet drugs and/or anticoagulants. Paraneoplastic syndromes (PNS) represent a frequent complication of renal cell carcinomas (RCC) and include different hematological symptoms in patients, whilst occlusion of arterial blood vessels displays a rare form of PNS accompanying renal tumors.

**Case presentation:**

We report the case of a 62-year old man who was initially hospitalized due to acute coronary syndrome. He subsequently underwent coronary angioplasty treatment including multiple stenting and treatment with ticagrelor and aspirin. Post-interventional, acute arterial thrombotic emboli of several limb arteries required thrombectomy. By computer tomography we identified a renal lesion suspicious for an RCC and suspected a PNS as underlying cause of the thrombotic complications. Triple anticoagulant therapy was maintained with therapeutic dose low molecular weight heparin (LMWH), aspirin, and clopidogrel, by which we replaced ticagrelor. Surgery was postponed for 4 weeks. We paused LMWH, aspirin and clopidogrel only at the day of surgery and perioperatively restored hemostasis by transfusion of two platelet concentrates. Laparoscopic nephrectomy was uneventful. Pathology confirmed a clear cell RCC. The patient fully recovered whilst slowly reducing anticoagulation dose.

**Conclusions:**

A multidisciplinary team approach of experts in urology, cardiology and hemostasis was key in managing this patient since a personalized thrombosis consult was needed to minimize the risk of reinfarction due to in-stent thrombosis. We report a therapeutic protocol that may be helpful for the management of similar cases. Furthermore, the finding of thrombotic arterial occlusions in larger blood vessels represents a novel complication of PNS in RCC and adds to the varied possible manifestations of this clinical chameleon.

## Background

Cancer induces a prothrombotic state. In about 25% of patients above the age of 50 years presenting with acute thrombosis, undiagnosed cancer is the underlying cause. Sometimes cancer is associated with massive hyper coagulopathy requiring removal of the tumor. Surgical treatment of cancer in a patient with recent thromboembolic complications is highly complex, as continuation of anticoagulation and antiplatelet therapy increases the bleeding risk, while cessation of these drugs increases the risk for re-thrombosis.

Renal cell carcinoma is the most common primary tumor of the kidney while it accounts for around 2–3% of all cancers worldwide [1]. Today it is most frequently diagnosed incidentally through radioimaging such as CT or MRI scans, whereas the classical symptom trias of haematuria, palpable tumor and flank pain leads to diagnosis in only 10% of patients [2]. Paraneoplastic syndromes are reported to occur in up to 68% of patients with RCC and can be subdivided into endocrinological and non-endocrinological forms, with hypertension, polycythaemia and hypercalcaemia being primary symptoms of manifestation [3]. Thus far, acute arterial occlusion has rarely been observed accompanying RCCs and has not been reported in larger peripheral blood vessels.

We report a patient who developed myocardial infarction and massive acute thrombotic emboli in main arteries due to a supposed paraneoplastic syndrome caused by an incidentally found clear cell RCC. We present the clinico-pathological outcome of an interdisciplinary approach.

## Case presentation

**Fig. 1:**
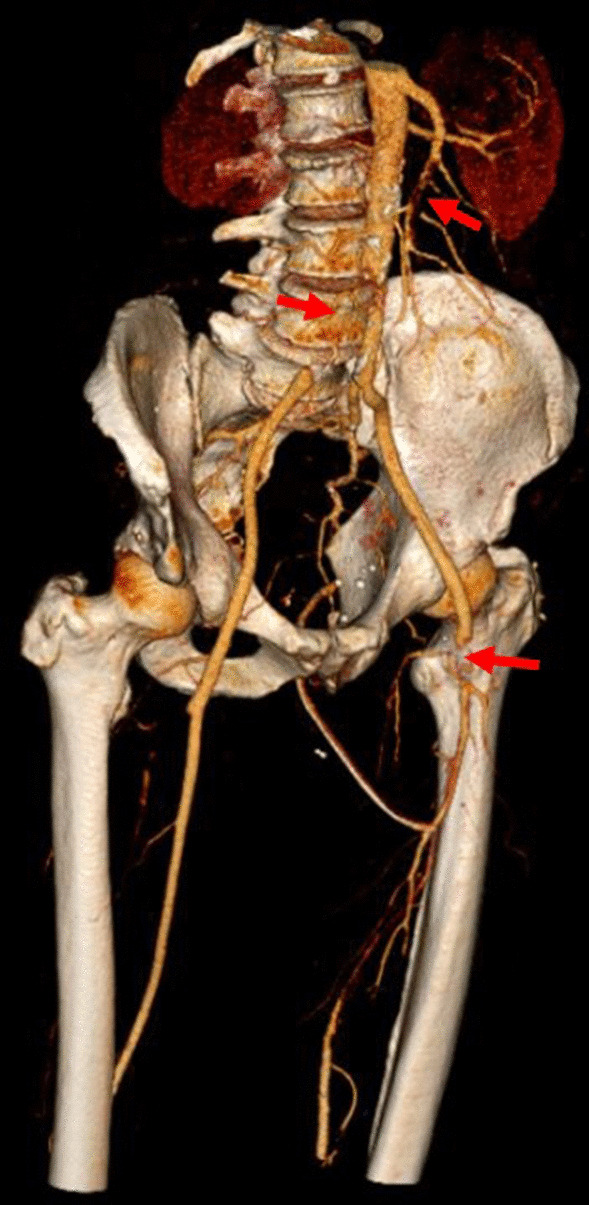
3D-Reconstruction of CT images showing several arterial thrombotic embolies with incomplete vascular occlusion. Red darts indicate a branch of the upper mesenterial artery, the right common iliac artery and the left femoral artery from top to bottom

**Fig. 2:**
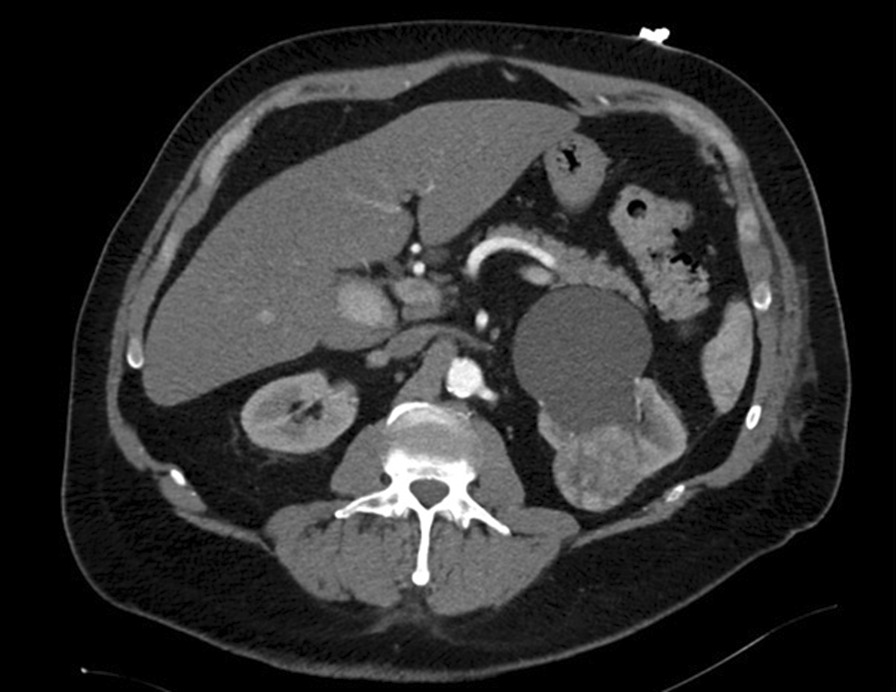
Abdominal CT scan showing a renal tumor of the left side with adjacent renal cyst

A 62-year old male with a history of metabolic syndrome including arterial hypertension, diabetes type 2, hyperlipidemia, obesity and sleep apnea syndrome was initially admitted to our hospital due to acute coronary syndrome. ST-elevated myocardial infarction was diagnosed followed by primary percutaneous coronary intervention (PCI) including angioplasty treatment and application of a singular drug-eluting stent in the right coronary artery. Post-interventional, dual anti-platelet therapy (DAPT) using ticagrelor and aspirin was initiated as well as thrombosis prophylaxis with LMWH. Initial blood testing was normal, particularly erythrocytosis, thrombocytosis and coagulopathy were excluded. As the patient remained instable, at day 3 a second PCI was performed and six drug-eluting stents placed in the left anterior descending artery. At day 10, the patient complained about severe pain in both legs. A thoracoabdominal CT scan showed several acute arterial thrombotic emboli, including incomplete occlusion of the right common iliac artery, the left femoral artery, several arteries of the lower leg and a main branch of the upper mesenteric artery (Fig. 1). Incidentally, the CT scan also showed a left renal tumor highly suspicious for a renal cell carcinoma, accompanied by a large renal cyst (Fig. 2). Platelet count was 149 Gpt/l and acute heparin-induced thrombocytopenia (HIT) was considered very unlikely [4].

Therapeutic anticoagulation using unfractionated heparin was started in addition to the ongoing antiplatelet therapy. As this did not result in improvement of the leg perfusion arteriectomy was performed removing most of the occluding material. Histo-pathology confirmed fresh thrombotic emboli. Due to the radiologic suspect of an RCC, a PNS as cause of the massive coagulopathy was suspected.

In a multidisciplinary conference including cardiology, thrombosis consult, vascular surgery and urology, following issues were defined:

The risk for in-stent thrombosis of the seven drug-eluting coronary stents was considered very high in the absence of effective DAPT.

Minimally invasive, surgical total nephrectomy was considered as the intervention with the lowest bleeding risk, which however could not be performed on triple therapy with heparin, ticagrelor and aspirin.

The triple therapy had resulted in a decrease of the D-dimer and stabilization of the platelet count. It was therefore decided to keep the patient on triple anticoagulation therapy for four weeks to allow beginning of endothelialization of the coronary stens and of the altered limb arteries. Ticagrelor is a reversible P2Y12 inhibitor, which is present in the circulation in high concentration. Although the half-life of the drug is only 8 h, it takes about 72–96 h until the high drug levels are decreased to a level, which allows platelet transfusion without inhibition of the transfused platelets [5]. We therefore switched ticagrelor to clopidogrel. Clopidogrel is a direct P2Y12 inhibitor. When it binds to platelets, platelet ADP receptor is irreversibly inhibited for the life span of the platelet, but the active metabolite of clopidogrel has a short half-life of only 1–2 h.

DAPT and LMWH (enoxaparin 1 mg per KG body weight b.i.d) were maintained for 4 weeks and platelet counts, D-dimer and anti-factor Xa were monitored on a weekly basis to assure sufficient anticoagulation. Aspirin, clopidogrel and LMWH were stopped 24 h before surgery. According to the Greifswald bridging protocol, two platelet concentrates were transfused immediately before surgery [6]. Laparoscopic surgery was then performed without bleeding complications. Aspirin and LMWH in prophylactic dose (dalteparin 5000 aFXaU) were restarted six hours after surgery and clopidogrel 48 h after surgery. On the second postoperative day dalteparin was escalated to 5000 aFXa b.i.d. (half therapeutic dose) and therapeutic dose LMWH resumed at day 5. Pathology confirmed clear cell renal cell carcinoma pT3a L0 V0 R0 G2. After an uneventful postsurgical course, the patient was discharged at day eight after surgery. In the post-hospital setting, clopidogrel and aspirin were maintained for one year as indicated after myocardial infarction, while LMWH was stepwise reduced under control of repeated D-Dimer blood testing to prophylactic dose and could eventually be terminated after 6 weeks.

## Discussion and conclusions

This patient exemplifies the dilemma of management of patients with newly diagnosed cancer complicated by acute thromboembolic complications, especially if the thrombotic state of the patient is induced by a paraneoplastic syndrome. In-stent thrombosis of the newly implanted coronary stents was considered to be of highest risk for myocardial infarction of this patient. As aggressive triple therapy with antiplatelet drugs and therapeutic dose anticoagulation stabilized the procoagulatory state, a wait and watch strategy for four weeks was considered acceptable. During this time control of anticoagulation was monitored by three parameters: D-dimer as an indicator for progression of fibrin formation, the platelet count as parameter for increased thrombin generation and consumptive coagulopathy, and aFXa levels for compliance and exclusion of consumption of heparin by acute phase proteins. As these parameters remained stable no dose escalation of LMWH was performed. Ticagrelor is recommended as first line P2Y12 inhibitor in patients with acute coronary syndrome. However, a major disadvantage of ticagrelor is its long presence in the circulation. After the last intake the drug persists for 72–96 h. As a small molecule it distributes not only in the intravascular space but also in the interstitial fluid from which it redistributes into the vasculature. Cessation of antiplatelet therapy for 72–96 h before surgery was inacceptable in this patient due to the high risk of in-stent or limb artery thrombosis four weeks after multiple arterial occlusions. Therefore, treatment was a switched from ticagrelor to clopidogrel (while aspirin and LMWH were maintained). Although clopidogrel inhibits platelets irreversible, its active metabolite has a short half-life. This allowed maintenance of antiplatelet therapy until 24 h before surgery, just skipping the morning dose at the day of surgery and subsequently restoring platelet function by platelet transfusion for the peri-interventional period. From on six hours after surgery antiplatelet and anticoagulatory treatment were restarted and escalated stepwise while the patient was carefully monitored for bleeding symptoms.

PNS in renal cell carcinoma are a common phenomenon with a wide variety of disease-associated symptoms [3, 7]. Among the many possible manifestations, vascular and haematological syndromes play a major role. Hypertension is a frequent symptom of PNS and is found in 7–35% of patients. It is assumed to have multiple causes such as combined polycythaemia, increased erythropoietin (EPO) expression or abnormal renin secretion of renal tumor mass [8]. A possible rare reason of acute emboli and stroke can be found in PNS-related polycythaemia which occurs in 8% of RCC patients due to elevated EPO blood levels produced by tumorous renal tissue [9]. Several other haematological abnormalities associated to PNS in RCC have been reported including thrombocytopenia or leukocytosis and are thought to be due to excessive tumor production of transmitters such as IL-6 or granulocyte colony stimulating factor [3].

Much more rarely, PNS-associated arterial ischemia and emboli have been reported in RCC. Few cases of acute digital ischaemia have been described showing classical symptoms of Raynaud’s phenomenon. In these patients, tumor progression is more aggressive compared to the average patient cohort [10]. In another report, a fatal outcome of pulmonary tumor thrombotic microangiopathy based on a papillary RCC was identified, in which tumorous microemboli formed in the pulmonary arterioles and subsequently lead to right heart failure [11]. Venous thrombosis in central retinal veins or the upper caval vein are further manifestations of PNS, probably aggravated by elevated levels of antiphospholipid and anticardiolipin antibodies [12, 13].

Besides the incidentally identified RCC, other possible etiologies of limb artery emboli are thrombosis at the site of femoral artery puncture during PCI. An antiphospholipid syndrome which occurs as PNS or HIT had been excluded during the acute phase by negative antibody tests. The authors view the fact of total symptom resolution after nephrectomy is partial proof for the role of the tumor in disease progression as it is typical for PNS [14]. Nevertheless, PNS remains an exclusion diagnosis and the assumption of it being the underlying cause of the depicted symptoms must be seen as speculative. Additional and more specific biochemical investigations to support the diagnosis of PNS except those mentioned have not been performed in this study.

In conclusion, PNS in renal cell carcinoma can be perceived as a clinical chameleon showing a wide variety of symptoms. Unexplained arterial thrombosis seems to be another manifestation of PNS. Although elimination of the primary cause by surgical tumor removal is most important, a multidisciplinary treatment approach is often required to manage the multiple complications of PNS.

## Data Availability

Data sharing is not applicable to this article as no datasets were generated or analysed during the current study.

## Abbreviations

DAPT: Dual anti-platelet therapy
EPO: Erythropoetin
HIT: Heparin-induced thromboyctopenia
LMWH: Low molecular weight heparine
PCI: Percutaneous coronary intervention
PNS: Paraneoplastic syndrome
RCC: Renal cell carcinoma
